# The correlation between high-density lipoprotein cholesterol and bone mineral density in adolescents: a cross-sectional study

**DOI:** 10.1038/s41598-023-32885-x

**Published:** 2023-04-08

**Authors:** Gao-Xiang Wang, Jun-Tong Li, De-Liang Liu, Shu-Fang Chu, Hui-Lin Li, Heng-Xia Zhao, Ze-Bin Fang, Wei Xie

**Affiliations:** 1 Department of Endocrinology, Shenzhen Traditional Chinese Medicine Hospital Affiliated to Nanjing University of Chinese Medicine, Shenzhen, 518033 Guangdong China; 2 Department of Endocrinology, Shenzhen Traditional Chinese Medicine Hospital, Shenzhen, 518033 Guangdong China; 3 The Fourth Clinical Medical College of Guangzhou University of Chinese Medicine, Shenzhen, 518033 Guangdong China

**Keywords:** Endocrinology, Medical research, Risk factors

## Abstract

Recent studies have shown a correlation between high-density lipoprotein cholesterol (HDL-C) and bone mineral density (BMD) in adults, but their relationship is unclear in adolescents. This study aimed to explore whether a correlation existed between them among adolescents aged 12–19. Data analyzed in our study was fetched from the National Health and Nutrition Examination Survey (NHANES) database 2011–2018. The relationship between HDL-C level and total BMD value was analyzed by multivariate logistic regression models, fitted smoothing curves, and generalized additive models. 3770 participants participated in this analysis. After adjusting for all relevant covariates involved in this study, we found a negative correlation between HDL-C levels and total bone density in male adolescents.Furthermore, the stratified analysis showed that all covariables-adjusted models retained the negative correlation excepting female, black, or Mexican American subgroups. An inverted U-shaped curve represented the correlation of HDL-C and total BMD among adolescents aged 16 to 19, and the turning point was 1.06 mmol/L. After adjusting for all relevant covariates involved in this study, the study found a negative correlation between HDL-C levels and total BMD in male adolescents aged 12 to 19, particularly among those of races other than Black and Mexican. There was a saturation effect between HDL-C level and total BMD in 16–19-year-old adolescents. The turning point was 1.06 mmol/L. Therefore, HDL-C might be a biomarker to detect bone health and further perform a more detailed examination.

## Introduction

Osteoporosis is a common skeleton disease characterized by decreased bone mass, bone fragility, and increased fracture risk^1^. It is noteworthy that osteoporosis affects not only elderly subjects or postmenopausal women, but more attention has been paid to adolescents and children^2^. In adolescence, which is a progressive ontogenesis period, skeleton microarchitecture and mineralization make a qualitative change, and 40% of the peak bone mass is accumulated in this window^3^. Making an exact assessment of adolescents’ skeletal health benefits their current and future quality of life. World Health Organization proposed bone mineral density (BMD) value tested using dual X-ray absorptiometry (DXA) is the gold standard to diagnose osteoporosis^4^. Identifying associated and potential detrimental factors for bone health is significant for preventing and detecting osteoporosis.

Serum lipids, which consist of many substances, play a crucial role in physiopathology and are strongly associated with various metabolic diseases. High-density lipoproteins-cholesterol (HDL-C) is considered beneficial cholesterol^5^. Higher HDL-C levels meant better cardiovascular health in the past^6^. However, some researchers have proposed different perspectives about HDL-C. Hamer et al. indicated that the high HDL-C also increased mortality in a large population-based sample^7^.

Numerous kinds of research revealed that lipids level was closely related to BMD value^8–10^. Some studies showed a negative correlation between HDL-C and adult BMD^11,12^. Despite Ibrahim Duran et al. demonstrated muscle mass inversely correlated with HDL-C level among adolescents^13^, no research clarified and defined the relationship between HDL-C level and BMD value in adolescents. Therefore, exploring the correlation of HDL-C concentration and BMD value would be beneficial for estimating the risk of osteoporosis, which could effectively promote the prevention and treatment of osteoporosis.

This study aimed to clarify the relationship between HDL-C and total BMD in adolescents aged 12–19. Population-based subjects were extracted from the National Health and Nutrition Examination Survey (NHANES) to provide credible evidence for this issue.

## Materials and methods

### Patient collection and data extraction

**Figure 1:**
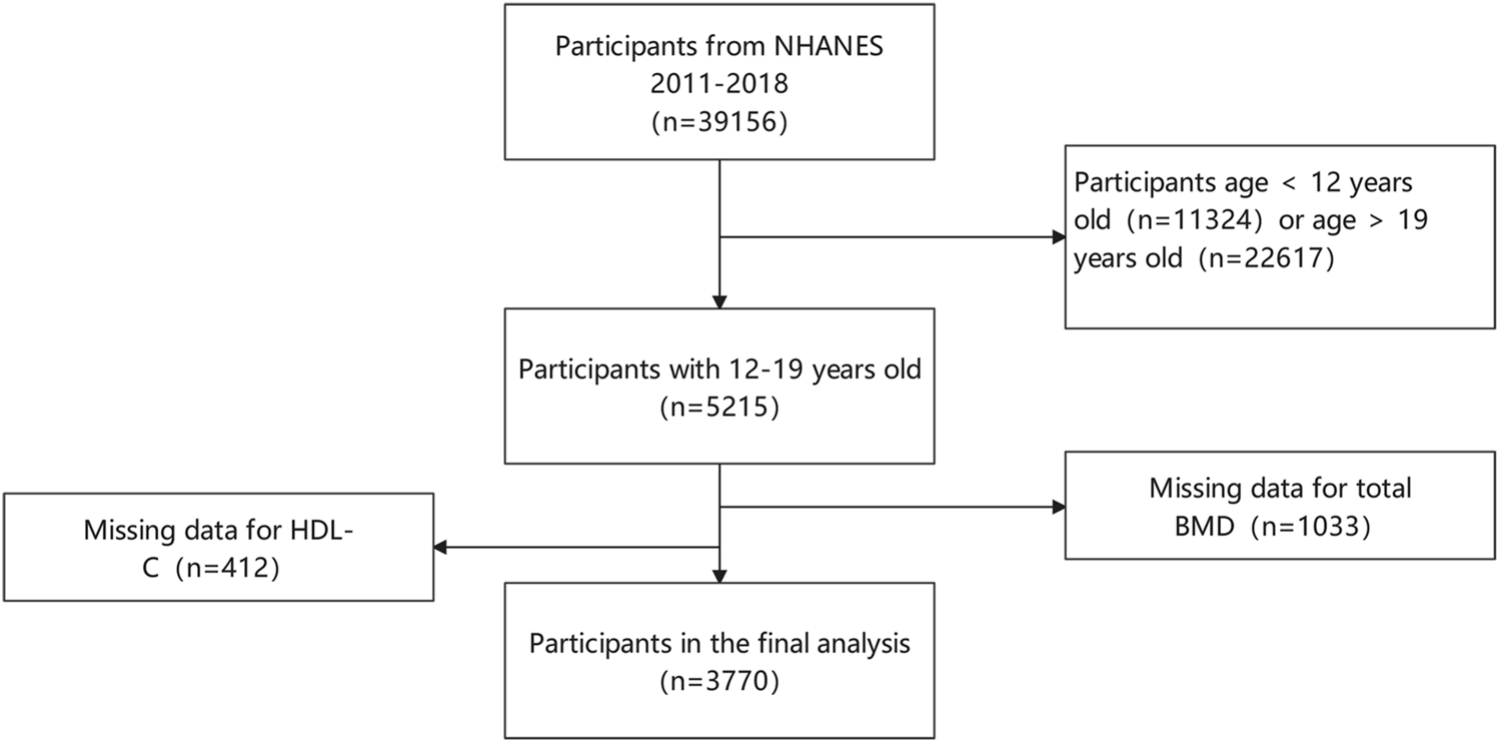
Workflow diagram of samples extraction from the NHANES database.

NHANES database is the largest population-based national survey in the world and is organized by the National Center for Health Statistics (NCHS) in the US. This survey is conducted every 2 years to evaluate the nutritional and health status of a representative of the noninstitutionalized US population. A complex stratified and multistage sampling design was applied to extract the representative population in the NHANES database. More details of the database are available at http://www.cdc.gov/nchs/nhanes/. The current study consisted of four data cycles (2011–2018). In this study, there were 39,156 participants enrolled from NHANES 2011–2018 database. After limiting to adolescents aged 12 to 19, the participants narrowed to 5215. Subjects with missing total BMD data (n = 1033) or HDL-C data (n = 412) were excluded. Finally, the data of 3770 cases were retained for analysis (Fig. 1). All NHANES protocols were ratified by the ethics review board of NCHS^14^.

### Covariates

Our study's dependent and independent variables were HDL-C and BMD, respectively. The Roche Modular P chemistry analyzer detected the HDL-C concentration or the Roche modular P and Roche Cobas 6000 chemistry analyzers in the 2011–2018 cycle. The BMD value of all cases in this study was tested by using dual-energy X-ray absorptiometry (DXA). Hologic Discovery Model A densitometer (Hologic, Inc., Bedford, Massachusetts) and Apex version 3.2 software were used to detect and analyze BMD. The subject was scanned, and their BMD was calculated using a whole-body scan. According to the relative regulations and guidelines of WHO, the mean BMD value of a young adult was a reference value.

Furthermore, demographic characteristics in the NHANES database were regarded as potential confounders, which were analyzed in this study. These covariates included categorical variables (age, gender, race) and continuous variables (ratio of family income to poverty and various physiological and biochemical indexes including total calcium (Tca), serum phosphorus (P), total protein (TP), total cholesterol (TC), alkaline phosphatase (ALP), serum uric acid (SUA), triglyceride (TG), glycohemoglobin (HbA1c), urinary albumin creatinine ratio (ACR), serum creatinine (Scr), and blood urea nitrogen (BUN).

### Statistical analysis

Based on the complex, multistage, stratified probability sampling design, sample weights were used in all statistical analyses through software R and Empower Stats. The study utilized weighted multivariate linear regression models to assess the correlation between HDL-C level and BMD value. Covariates were regulated as possible effect modulators. Therefore, we established three models for statistical inference in the subgroup analyses. Model 1 was adjusted with no covariates. Three covariates (age, gender, and race) were adjusted in model 2. In model 3, the ratio of family income to poverty, Tca, P, TP, ALP, TC, TG, HbA1c, Scr, ACR, SUA, and BUN were further adjusted. If the association of HDL-C with BMD was nonlinear, smooth curve fittings were applied to address the nonlinearity in these three models. Percentage and means ± standard deviation represented the categorical and continuous variables, respectively. Categorical variables adopted weighted chi-square tests, and continuous variables adopted weighted linear regression models to evaluate differences between groups.

### Ethics approval and consent to participate

Participants in NHANES must sign an informed consent form, the data is now publicly available, and the National Center for Health Statistics Ethics Review Board evaluates and authorizes it. Converting data into a format that can be analyzed is already feasible. The research methodology will be based on all statistics. All research will conform to applicable laws and standards when following the research's data usage guidelines.

## Results

### Characteristics of the study participants

Table 1 describes the weighted sociodemographic and medical characteristics of 3770 eligible participants. The variables, including Tca, P, ALP, TC, TG, Scr, SUA,TP, HDL-C, ACR, BUN,and total BMD, were significantly different among different gender groups.

**Table 1:** Weighted characteristics of the sample in this study.

	Male (n = 1979)	Female (n = 1791)	*P* value
Age (years)	15.41 ± 2.29	15.46 ± 2.20	0.4569
Race/ethnicity (%)			0.7132
White	54.11	54.69	
Black	13	12.02	
Mexican American	16.02	15.52	
Other race	16.87	17.77	
The ratio of family income to poverty (%)	2.44 ± 1.56	2.38 ± 1.59	0.2485
Total calcium (mmol/L)	2.41 ± 0.07	2.38 ± 0.08	< 0.0001
Serum phosphorus (mmol/L)	1.44 ± 0.23	1.34 ± 0.18	< 0.0001
Alkaline phosphatase (µ/L)	173.40 ± 105.62	98.75 ± 60.54	< 0.0001
Total cholesterol (mmol/L)	3.94 ± 0.72	4.13 ± 0.77	< 0.0001
Triglyceride (mmol/L)	1.17 ± 0.83	1.04 ± 0.63	< 0.0001
Glycohemoglobin (%)	5.23 ± 0.34	5.23 ± 0.40	0.9986
Serum creatinine (µmol/L)	68.94 ± 15.58	58.50 ± 11.53	< 0.0001
Serum uric acid (µmol/L)	333.45 ± 68.56	265.46 ± 58.05	< 0.0001
Urinary albumin creatinine ratio (mg/g)	20.31 ± 86.14	29.70 ± 110.33	0.0035
Blood urea nitrogen (mmol/L)	4.29 ± 1.24	3.74 ± 1.16	< 0.0001
Total protein (g/L)	72.54 ± 4.13	72.11 ± 4.01	0.0013
HDL cholesterol (mmol/L)	1.29 ± 0.30	1.39 ± 0.31	< 0.0001
Total bone mineral density (g/cm^2^)	1.04 ± 0.13	1.02 ± 0.10	< 0.0001

Continuous variables were represented via mean ± SD, calculated using a weighted linear regression model.

Categorical variables were presented via %. Calculated by The chi-square test.

### Associations between HDL-C and total BMD

**Figure 2:**
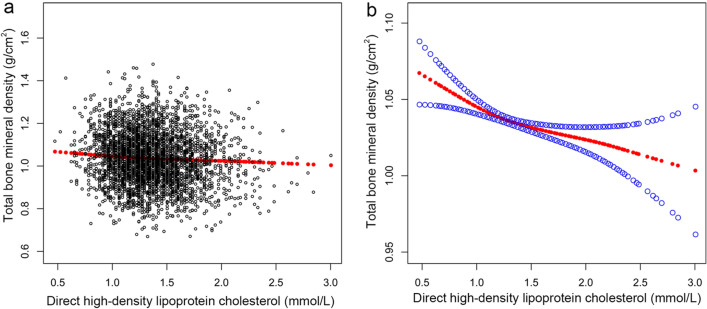
The expounded curved line correlation between the level of HDL-C and total BMD. (**a**) One black point was equal to a subject. (**b**) A 95% CI is expressed as an area between two blue dotted lines. Each point shows HDL-C magnitude, and a continuous line is drawn.

**Figure 3:**
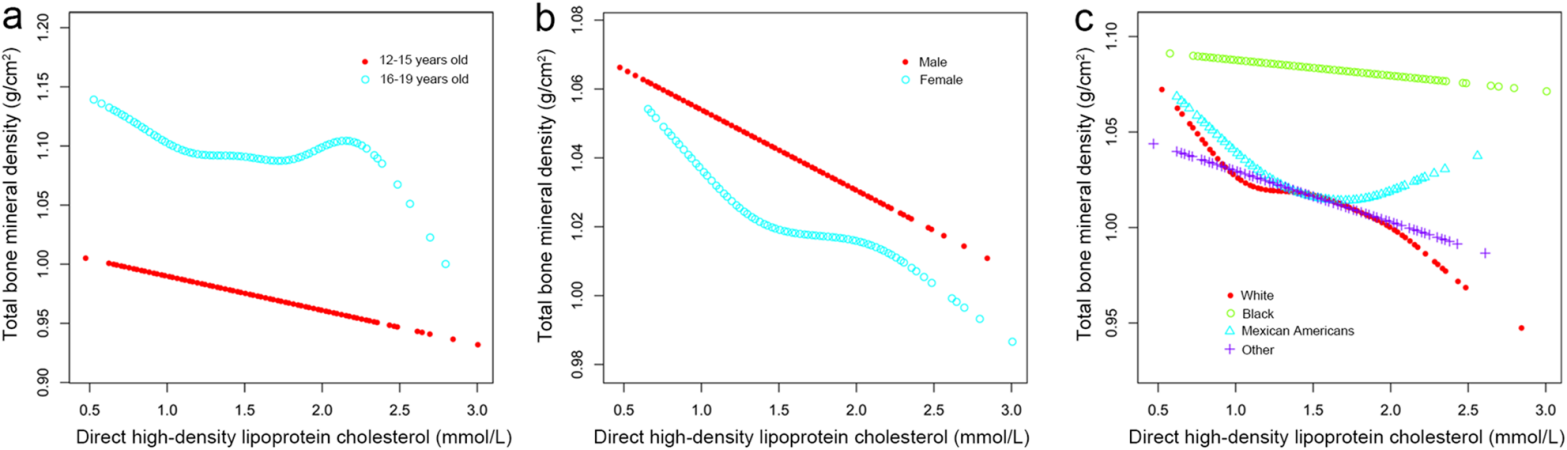
The HDL-C concentration and BMD relationship. (**a**) The illustrated line was stratified by age. (**b**) The illustrated line was stratified by sex. (**c**) The illustrated line was stratified by race/ethnicity.

The multivariate regression analysis to reveal the association of total BMD with HDL-C was presented in Table 2. An inversive association existed between HDL-C level and total BMD value in model 1 [− 0.047 (− 0.059, − 0.035)] with no variables adjusted, model 2 [− 0.038 (− 0.048, − 0.028)] with age, gender and race were adjusted, and model 3 [− 0.025 (− 0.036, − 0.014)] with all potential covariables were adjusted. Furthermore, there were three stratified analysis (stratified by age, gender, or race) that indicated the correlation was not significant in female adolescents [− 0.015 (− 0.029, 0.000)], black subjects [− 0.005 (− 0.028, 0.017)] and Mexican American subjects [− 0.018 (− 0.044, 0.007)]. The current study adopted smooth curve fittings to present the nonlinear correlation of HDL-C and BMD (Fig. 2). Moreover, the results of the stratified analysis are visualized with generalized additive models in Fig. 3. In age-stratified analysis, among adolescents aged 16 to 19, an inverted U-shaped curve represented the correlation of HDL-C and total BMD (Fig. 3a).

**Table 2:** Association between high-density lipoprotein cholesterol and total bone mineral density.

Exposure	Model 1, β (95% CI)	Model 2, β (95% CI)	Model 3, β (95% CI)
Direct HDL cholesterol (mmol/L)	− 0.047 (− 0.059, − 0.035)	− 0.038 (− 0.048, − 0.028)	− 0.025 (− 0.036, − 0.014)
Quintiles of direct HDL cholesterol (mmol/L)			
Q1	Reference	Reference	Reference
Q2	− 0.019 (− 0.029, − 0.008)	− 0.008 (− 0.017, 0.001)	− 0.004 (− 0.012, 0.004)
Q3	− 0.015 (− 0.026, − 0.004)	− 0.011 (− 0.020, − 0.003)	− 0.005 (− 0.013, 0.004)
Q4	− 0.038 (− 0.049, − 0.027)	− 0.028 (− 0.037, − 0.019)	− 0.015 (− 0.025, − 0.006)
* P* for trend	< 0.001	< 0.001	0.002
Stratified by age			
12–15 years old	− 0.043 (− 0.058, − 0.028)	− 0.054 (− 0.069, − 0.039)	− 0.022 (− 0.038, − 0.007)
16–19 years old	− 0.036 (− 0.051, − 0.021)	− 0.019 (− 0.033, − 0.004)	− 0.023 (− 0.039, − 0.007)
Stratified by gender			
Male	− 0.071 (− 0.090, − 0.051)	− 0.043 (− 0.058, − 0.029)	− 0.036 (− 0.053, − 0.020)
Female	− 0.016 (− 0.031, − 0.000)	− 0.024 (− 0.037, − 0.010)	− 0.015 (− 0.029, 0.000)
Stratified by race			
White	− 0.054 (− 0.077, − 0.030)	− 0.040 (− 0.059, − 0.021)	− 0.030 (− 0.051, − 0.009)
Black	− 0.058 (− 0.083, − 0.032)	− 0.030 (− 0.051, − 0.008)	− 0.005 (− 0.028, 0.017)
Mexican American	− 0.044 (− 0.070, − 0.018)	− 0.030 (− 0.052, − 0.008)	− 0.018 (− 0.044, 0.007)
Other race	− 0.062 (− 0.085, − 0.039)	− 0.044 (− 0.064, − 0.024)	− 0.036 (− 0.057, − 0.014)

Model 1: No adjusted.

Model 2: Adjusted age, gender, and race.

Model 3: Adjusted all covariates were.

The stratification variable itself was not adjusted in the subgroup analysis.

When stratified according to gender and age (Table 3), we found that after adjusting all variables, there was a negative correlation between HDL-C and total BMD in male adolescents of all ages but not in female adolescents. When stratified according to gender and race, we found that after adjusting all variables, there was a negative correlation between HDL-C and total BMD between white men and people of other races. Additionally, Table 4 of this study reveals a saturation effect analysis between HDL-C and total BMD. The results indicate a saturation effect was observed in adolescents aged 16 to 19. The turning point of direct HDL-C was the same in males and females (1.06 mmol/L). However, we did not find this effect in 12–16 year-old teenagers.

**Table 3:** Association between high-density lipoprotein cholesterol and total bone mineral density stratified by gender, age, and race.

Stratified by age	Male	Female
Stratified by age		
12–15 years old		
Model 1, β (95% CI)	− 0.060 (− 0.082, − 0.039) < 0.00001	− 0.027 (− 0.048, − 0.005) 0.01517
Model 2, β (95% CI)	− 0.071 (− 0.092, − 0.050) < 0.00001	− 0.032 (− 0.053, − 0.011) 0.00258
Model 3, β (95% CI)	− 0.034 (− 0.055, − 0.013) 0.00129	− 0.011 (− 0.032, 0.010) 0.31799
16–19 years old		
Model 1, β (95% CI)	− 0.007 (− 0.031, 0.017) 0.57234	− 0.016 (− 0.034, 0.003) 0.09634
Model 2, β (95% CI)	0.016 (− 0.040, 0.008) 0.19389	− 0.021 (− 0.039, − 0.003) 0.02354
Model 3, β (95% CI)	− 0.027 (− 0.053, − 0.001) 0.04248	− 0.020 (− 0.042, 0.001) 0.05890
Stratified by race		
White		
Model 1, β (95% CI)	− 0.094 (− 0.131, − 0.057) < 0.00001	− 0.013 (− 0.042, 0.017) 0.40100
Model 2, β (95% CI)	− 0.060 (− 0.087, − 0.033) 0.00002	− 0.014 (− 0.039, 0.011) 0.28524
Model 3, β (95% CI)	− 0.054 (− 0.085, − 0.024) 0.00050	− 0.008 (− 0.036, 0.021) 0.60236
Black		
Model 1, β (95% CI)	− 0.067 (− 0.107, − 0.026) 0.00126	− 0.045 (− 0.077, − 0.014) 0.00465
Model 2, β (95% CI)	0.000 (− 0.031, 0.031) 0.99446	− 0.044 (− 0.072, − 0.016) 0.00218
Model 3, β (95% CI)	0.013 (− 0.021, 0.047) 0.44398	− 0.021 (− 0.050, 0.009) 0.16453
Mexican American		
Model 1, β (95% CI)	− 0.039 (− 0.081, 0.002) 0.06555	− 0.036 (− 0.068, − 0.005) 0.02434
Model 2, β (95% CI)	− 0.027 (− 0.060, 0.006) 0.10693	− 0.032 (− 0.060, − 0.004) 0.02394
Model 3, β (95% CI)	− 0.026 (− 0.066, 0.013) 0.19245	− 0.023 (− 0.057, 0.011) 0.19028
Other race		
Model 1, β (95% CI)	− 0.088 (− 0.125, − 0.051) < 0.00001	− 0.016 (− 0.045, 0.012) 0.25417
Model 2, β (95% CI)	− 0.039 (− 0.069, − 0.009) 0.01086	− 0.030 (− 0.056, − 0.004) 0.02367
Model 3, β (95% CI)	− 0.044 (− 0.076, − 0.012) 0.00695	− 0.023 (− 0.053, 0.006) 0.12544

Model 1: No adjusted.

Model 2: Age and race were adjusted.

Model 3: All covariates were adjusted.

The stratification variable itself was not adjusted in the subgroup analysis,

**Table 4:** Saturation effect analysis of high-density lipoprotein cholesterol and total bone mineral density.

Direct HDL cholesterol (mmol/L)	Male	Female
	β (95% CI), P-value	β (95% CI), P-value
12–15 years old		
Fitting by the standard linear model	− 0.032 (− 0.052, − 0.012) 0.0022	− 0.012 (− 0.033, 0.009) 0.2507
Fitting by the two-piecewise linear model		
Turn point of direct HDL cholesterol (mmol/L)	0.93	0.98
< K, effect1	− 0.158 (− 0.312, − 0.004) 0.0450	0.104 (− 0.112, 0.320) 0.3463
> K, effect2	− 0.030 (− 0.050, − 0.009) 0.0045	− 0.015 (− 0.036, 0.006) 0.1712
The effect difference	0.128 (− 0.027, 0.283) 0.1066	− 0.119 (− 0.339, 0.101) 0.2906
The predicted value of the equation at the folding point	0.983 (0.972, 0.995)	0.994 (0.983, 1.006)
Log-likelihood ratio test	0.103	0.285
16–19 years old		
Fitting by the standard linear model	− 0.028 (− 0.054, − 0.002) 0.0342	− 0.019 (− 0.040, 0.002) 0.0780
Fitting by the two-piecewise linear model		
Turn point of direct HDL cholesterol (mmol/L)	1.06	1.06
< K, effect1	− 0.129 (− 0.216, − 0.041) 0.0042	− 0.232 (− 0.344, − 0.120) < 0.0001
> K, effect2	− 0.009 (− 0.040, 0.021) 0.5480	− 0.003 (− 0.025, 0.020) 0.8228
The effect difference	0.119 (0.020, 0.219) 0.0192	0.229 (0.111, 0.348) 0.0002
The predicted value of the equation at the folding point	1.118 (1.108, 1.129)	1.058 (1.047, 1.068)
Log-likelihood ratio test	0.018	< 0.001

All covariates were adjusted.

## Discussion

We explored the relationship between HDL-C and BMD in adolescents. A higher quality of skeleton in adolescence protects against osteoporosis later in life^15^. Multivariate logistic regression analysis indicated an inversive correlation between HDL-C concentration with total BMD value. HDL-C was well known as beneficial cholesterol, promoting reversed cholesterol transportation and decreasing the incidence of various chronic diseases, including cardiovascular disease and endocrine disorders^16,17^. However, more and more researchers suggested the protective effect of HDL-C in some diseases was overestimated^7,18^. Previous research has confirmed that the correlation of HDL-C with muscle mass was also negative among adolescents^13,19^. Therefore, HDL-C level might be a potential biomarker for completing the evaluation of skeleton health in adolescents and improving quality of life in the future.

The relationship between HDL-C level and BMD value had been widely studied, but a unanimous agreement was not reached. Yuchen Tang et al. showed that HDL-C levels were inversely correlated with BMD value and might predict bone loss and osteoporosis^20^. Qi Zhang et al. explored the relationship of serum cholesterols (HDL-C, LDL-C, and TC) with lumber BMD. These three cholesterols indicated an inversive association with BMD in Chinese postmenopausal participants^12^. Peng Niu et al. confirmed the inverse correlation of HDL-C level and BMD through their analysis of the MIDUS II study^21^. However, Irene Zolfaroli et al. revealed that HDL-C level, but not TC and LDL-C, was positively relevant to the femoral neck and lumbar BMD^22^. Song-Seng Loke et al. suggested a positive association existed in Taiwanese older women, which was a reverse correlation in men. This finding reminded the importantce of gender^23^. Furthermore, Lian-Hua Cui et al. recruited 730 rural pre- or postmenopausal women and revealed no significant connection between HDL-C and BMD^24^. The reasons for controversial issues in previous studies may include small sample sizes, insufficient scientific data collection process, different populations analyzed, and differences in adjusted covariates.These controversial conclusions prompted us to develop more exploration, and the current study was more plausible. Firstly, 4 cycles of data were extracted from the NHANES database, which provided a larger sample. Second, the study considered gender, which might be a possible impact. Adolescents, not only females, were included in the study. Third, more potential variables that influence total BMD were adjusted. We found a negative association between HDL-C and BMD in a specific population of adolescents. This result enriches the research field more detailed and comprehensive to provide a more substantial reference for clinical application.

In this study, after adjusting all variables, the female subgroup did not have an association between HDL-C and BMD. Previous research had also suggested that gender hormones were possibly influencing factors in HDL-C's and BMD's correlation^25–27^. Ke Xu et al. investigated the relationship between gender hormones with BMD value. They demonstrated that the relationship between sex hormones such as testosterone, estradiol, or sex hormone-binding globulin levels and BMD were opposite in the boy and girl subgroups^28^. In summary, sex hormones might be the crucial cause of this association. In addition, a saturation analysis showed an inverted U-shaped curve representing the correlation between HDL-C and total BMD among adolescents aged 16 to 19, stratified by gender and age. The turning point was 1.06 mmol/L for both male and female adolescents. This variation tendency could be explained by a dramatic change in metabolic status, environmental risk factors, and other variables.

## Limitation and strengths

Recognizing the correlation between HDL-C and BMD could help physicians better evaluate bone health status.The advantages of this study are the relatively large sample size, the use of data from public databases which ensures scientific data collection, and gender and age stratified analysis. however, there were some limitations in this study. (i) All of the included participants were American. The inversive association might be inconsistent in other countries. (ii) Even though research had revealed that the sex hormones were influencing factors of the correlation, the NHANES databases did not contain detailed information about these factors. (iii) A cross-sectional design cannot confirm the cause-effect connection. Researchers should conduct more prospective studies to solve the causality.

## Conclusion

This study indicated a negative association between HDL-C and total BMD values among male adolescents. Therefore, adolescents with higher HDL-C concentrations were needed to monitor the value of BMD and prevent bone loss in the future.

## Data Availability

Data from the survey is publicly available online at http://www.cdc.gov/nchs/nhanes/ for data users worldwide.
